# 3-Bromo-6-nitro-1-(prop-2-yn­yl)-1*H*-indazole

**DOI:** 10.1107/S1600536811046927

**Published:** 2011-11-12

**Authors:** Nabil El Brahmi, Mohamed Benchidmi, El Mokhtar Essassi, Sonia Ladeira, Seik Weng Ng

**Affiliations:** a Laboratoire de Chimie Organique Hétérocyclique, Pôle de Compétences Pharmacochimie, Université Mohammed V-Agdal, BP 1014 Avenue Ibn Batout, Rabat, Morocco; b Laboratoire de Chimie de Coordination, route de Narbonne, 31077 Toulouse, France; c Department of Chemistry, University of Malaya, 50603 Kuala Lumpur, Malaysia; d Chemistry Department, King Abdulaziz University, PO Box 80203 Jeddah, Saudi Arabia

## Abstract

In the title compound, C_10_H_6_BrN_3_O_2_, the indazole fused-ring system is nearly planar (r.m.s. deviation = 0.008 Å); its nitro substituent is nearly coplanar with the fused ring [dihedral angle = 4.5 (2)°]. In the crystal, adjacent mol­ecules are linked by weak acetyl­ene–nitro C—H⋯O hydrogen bonds, generating a helical chain running along the *b* axis.

## Related literature

For a related compound, 1-allyl-3-chloro-6-nitro-1*H*-indazole, see: El Brahmi *et al.* (2009 ▶).
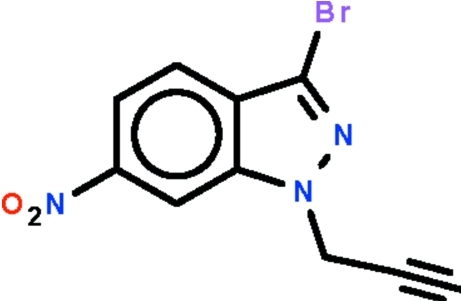


## Experimental

### 

#### Crystal data

C_10_H_6_BrN_3_O_2_
*M* *_r_* = 280.09
Monoclinic, 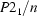

*a* = 14.6573 (3) Å
*b* = 4.1650 (1) Å
*c* = 17.4566 (3) Å
β = 102.659 (1)°
*V* = 1039.78 (4) Å^3^
*Z* = 4
Mo *K*α radiation
μ = 3.94 mm^−1^
*T* = 295 K
0.50 × 0.10 × 0.05 mm

#### Data collection

Bruker APEX DUO diffractometer
Absorption correction: multi-scan (*SADABS*; Sheldrick, 1996 ▶) *T*_min_ = 0.243, *T*_max_ = 0.827
14908 measured reflections
3137 independent reflections
2236 reflections with *I* > 2σ(*I*)
*R*_int_ = 0.023

#### Refinement

*R*[*F* ^2^ > 2σ(*F* ^2^)] = 0.028
*wR*(*F* ^2^) = 0.079
*S* = 1.03
3137 reflections
149 parameters
H atoms treated by a mixture of independent and constrained refinement
Δρ_max_ = 0.52 e Å^−3^
Δρ_min_ = −0.76 e Å^−3^

### 

Data collection: *APEX2* (Bruker, 2009 ▶); cell refinement: *SAINT* (Bruker, 2009 ▶); data reduction: *SAINT*; program(s) used to solve structure: *SHELXS97* (Sheldrick, 2008 ▶); program(s) used to refine structure: *SHELXL97* (Sheldrick, 2008 ▶); molecular graphics: *X-SEED* (Barbour, 2001 ▶); software used to prepare material for publication: *publCIF* (Westrip, 2010 ▶).

## Supplementary Material

Crystal structure: contains datablock(s) global, I. DOI: 10.1107/S1600536811046927/xu5386sup1.cif

Structure factors: contains datablock(s) I. DOI: 10.1107/S1600536811046927/xu5386Isup2.hkl

Supplementary material file. DOI: 10.1107/S1600536811046927/xu5386Isup3.cml

Additional supplementary materials:  crystallographic information; 3D view; checkCIF report

## Figures and Tables

**Table 1:** Hydrogen-bond geometry (Å, °)

*D*—H⋯*A*	*D*—H	H⋯*A*	*D*⋯*A*	*D*—H⋯*A*
C10—H1⋯O1^i^	0.96 (3)	2.45 (3)	3.399 (3)	167 (3)

Symmetry code: (i) 
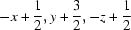
.

**Fig. 1.**
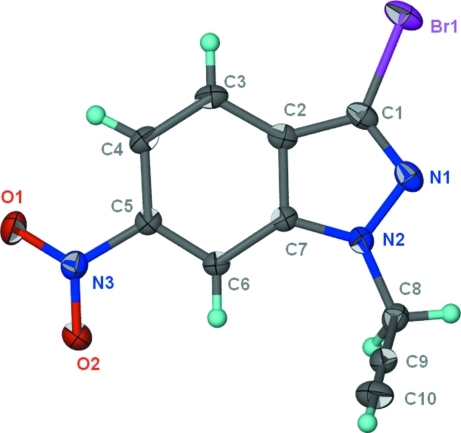
Thermal ellipsoid plot (Barbour, 2001) of C10H6BrN3O2 at the 50% probability level; hydrogen atoms are drawn as spheres of arbitrary radius.

**Fig. 2.**
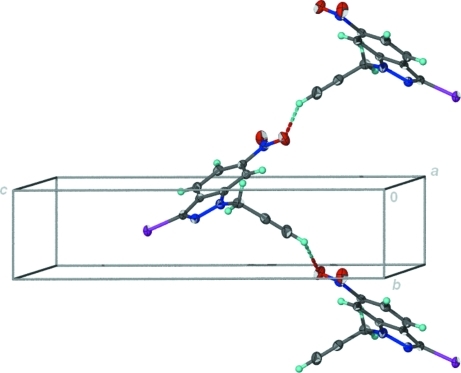
Helical chain motif.

## supplementary crystallographic information

### Comment

We reported 1-allyl-3-chloro-6-nitro-1*H*-indazole, which exists as two independent molecules (El Brahmi *et al.*, 2009). The present 1-propynyl-3-bromo-6-nitro-1*H*-indazole (Scheme I) also has halogen subsituent in the same position but the asymmetric unit consists of one molecule only. The indazole fused-ring is planar; its nitro substituent is nearly coplanar with the fused ring (Fig 1.). Adjacent molecules are linked by a C–H_acetylene_···O_nitro_ hydrogen bond to generate a helical polymer running along the *b*-axis of the monoclinic unit cell (Fig. 2). Weak Br···Br contacts of 3.57 Å are present.

### Experimental

3-Bromo-6-nitroindazole (1.2 g, 5 mmol) and propargyl bromide (1.2 g, 10 mmol) were reacted in THF (40 ml) in the presence of potassium carbonate (1.4 g, 10 mmol) and tetra-*n*-butylammonium bromide (0.5 mmol). The mixture was stirred for 24 h, filtered, and the THF removed under vacuum. The product was separated by chromatography on silica gel with a hexane:ethyl acetate (9:1) solvent system. The compound was obtained as yellow crystals.

### Refinement

Carbon-bound H-atoms were placed in calculated positions (C–H 0.93 Å) and were included in the refinement in the riding model approximation, with *U*(H) set to 1.2*U*(C). The acetylenic H-atom was located in a difference Fourier map and was refined. The 1 0 1 and -1 0 1 reflections were omitted owing to bad agreement.

### Crystal data

**Table:**

C_10_H_6_BrN_3_O_2_	*F*(000) = 552
*M**_r_* = 280.09	*D*_x_ = 1.789 Mg m^−^^3^
Monoclinic, *P*2_1_/*n*	Mo *K*α radiation, λ = 0.71073 Å
Hall symbol: -P 2yn	Cell parameters from 5822 reflections
*a* = 14.6573 (3) Å	θ = 2.4–30.3°
*b* = 4.1650 (1) Å	µ = 3.94 mm^−^^1^
*c* = 17.4566 (3) Å	*T* = 295 K
β = 102.659 (1)°	Plate, yellow
*V* = 1039.78 (4) Å^3^	0.50 × 0.10 × 0.05 mm
*Z* = 4	

### Data collection

**Table:**

Bruker APEX DUO diffractometer	3137 independent reflections
Radiation source: fine-focus sealed tube	2236 reflections with *I* > 2σ(*I*)
graphite	*R*_int_ = 0.023
ω scans	θ_max_ = 30.5°, θ_min_ = 2.9°
Absorption correction: multi-scan (*SADABS*; Sheldrick, 1996)	*h* = −15→20
*T*_min_ = 0.243, *T*_max_ = 0.827	*k* = −5→5
14908 measured reflections	*l* = −24→24

### Refinement

**Table:**

Refinement on *F*^2^	Primary atom site location: structure-invariant direct methods
Least-squares matrix: full	Secondary atom site location: difference Fourier map
*R*[*F*^2^ > 2σ(*F*^2^)] = 0.028	Hydrogen site location: inferred from neighbouring sites
*wR*(*F*^2^) = 0.079	H atoms treated by a mixture of independent and constrained refinement
*S* = 1.03	*w* = 1/[σ^2^(*F*_o_^2^) + (0.0381*P*)^2^ + 0.4798*P*] where *P* = (*F*_o_^2^ + 2*F*_c_^2^)/3
3137 reflections	(Δ/σ)_max_ = 0.001
149 parameters	Δρ_max_ = 0.52 e Å^−^^3^
0 restraints	Δρ_min_ = −0.76 e Å^−^^3^

### Fractional atomic coordinates and isotropic or equivalent isotropic displacement parameters (Å^2^)

**Table:**

	*x*	*y*	*z*	*U*_iso_*/*U*_eq_	
Br1	0.234567 (17)	0.49344 (5)	0.664925 (11)	0.03944 (9)	
O1	0.44171 (11)	−0.5183 (4)	0.38032 (10)	0.0431 (4)	
O2	0.30475 (11)	−0.5312 (3)	0.30395 (9)	0.0401 (4)	
N1	0.11630 (11)	0.3405 (4)	0.52285 (9)	0.0296 (3)	
N2	0.11523 (10)	0.1781 (4)	0.45461 (9)	0.0262 (3)	
N3	0.35862 (11)	−0.4437 (4)	0.36351 (10)	0.0278 (3)	
C1	0.20201 (14)	0.3105 (4)	0.56494 (10)	0.0281 (4)	
C2	0.26030 (13)	0.1289 (4)	0.52666 (10)	0.0250 (4)	
C3	0.35342 (14)	0.0226 (4)	0.54510 (11)	0.0287 (4)	
H3	0.3930	0.0758	0.5927	0.034*	
C4	0.38431 (13)	−0.1624 (4)	0.49070 (11)	0.0278 (4)	
H4	0.4456	−0.2367	0.5011	0.033*	
C5	0.32278 (12)	−0.2386 (4)	0.41932 (10)	0.0237 (3)	
C6	0.23102 (12)	−0.1400 (4)	0.39812 (10)	0.0231 (3)	
H6	0.1923	−0.1923	0.3501	0.028*	
C7	0.20061 (12)	0.0457 (4)	0.45476 (11)	0.0226 (3)	
C8	0.02773 (13)	0.1524 (5)	0.39593 (11)	0.0288 (4)	
H8A	−0.0229	0.2344	0.4179	0.035*	
H8B	0.0150	−0.0722	0.3831	0.035*	
C9	0.03026 (13)	0.3299 (5)	0.32386 (11)	0.0293 (4)	
C10	0.03171 (17)	0.4760 (5)	0.26605 (13)	0.0403 (5)	
H1	0.033 (2)	0.595 (7)	0.2189 (19)	0.069 (9)*	

### Atomic displacement parameters (Å^2^)

**Table:**

	*U*^11^	*U*^22^	*U*^33^	*U*^12^	*U*^13^	*U*^23^
Br1	0.06481 (17)	0.03278 (12)	0.02030 (10)	0.00030 (9)	0.00838 (9)	−0.00163 (7)
O1	0.0298 (8)	0.0620 (11)	0.0371 (8)	0.0170 (7)	0.0068 (6)	−0.0001 (7)
O2	0.0352 (8)	0.0498 (9)	0.0340 (8)	0.0039 (6)	0.0049 (6)	−0.0132 (6)
N1	0.0398 (9)	0.0261 (8)	0.0257 (8)	0.0012 (7)	0.0131 (7)	0.0006 (6)
N2	0.0266 (8)	0.0284 (8)	0.0244 (7)	0.0018 (6)	0.0072 (6)	−0.0018 (6)
N3	0.0270 (8)	0.0301 (8)	0.0269 (8)	0.0028 (6)	0.0071 (6)	0.0046 (6)
C1	0.0415 (11)	0.0236 (8)	0.0199 (8)	−0.0026 (7)	0.0084 (7)	0.0014 (6)
C2	0.0319 (10)	0.0205 (8)	0.0220 (8)	−0.0023 (7)	0.0045 (7)	0.0034 (6)
C3	0.0310 (10)	0.0299 (9)	0.0220 (8)	−0.0045 (7)	−0.0012 (7)	0.0030 (7)
C4	0.0227 (9)	0.0292 (9)	0.0292 (9)	0.0002 (7)	0.0008 (7)	0.0053 (7)
C5	0.0245 (9)	0.0228 (8)	0.0241 (8)	−0.0010 (6)	0.0057 (7)	0.0039 (6)
C6	0.0238 (9)	0.0232 (8)	0.0218 (8)	−0.0023 (7)	0.0036 (6)	0.0012 (6)
C7	0.0235 (8)	0.0204 (8)	0.0236 (8)	−0.0016 (6)	0.0044 (7)	0.0029 (6)
C8	0.0239 (9)	0.0292 (9)	0.0335 (10)	−0.0003 (7)	0.0062 (7)	−0.0008 (7)
C9	0.0246 (9)	0.0323 (10)	0.0298 (9)	−0.0007 (7)	0.0032 (7)	−0.0060 (7)
C10	0.0429 (12)	0.0481 (13)	0.0283 (10)	−0.0086 (10)	0.0042 (9)	−0.0021 (9)

### Geometric parameters (Å, °)

**Table:**

Br1—C1	1.8682 (17)	C3—H3	0.9300
O1—N3	1.228 (2)	C4—C5	1.405 (2)
O2—N3	1.216 (2)	C4—H4	0.9300
N1—C1	1.315 (2)	C5—C6	1.377 (2)
N1—N2	1.367 (2)	C6—C7	1.403 (2)
N2—C7	1.367 (2)	C6—H6	0.9300
N2—C8	1.459 (2)	C8—C9	1.467 (3)
N3—C5	1.476 (2)	C8—H8A	0.9700
C1—C2	1.414 (3)	C8—H8B	0.9700
C2—C7	1.407 (2)	C9—C10	1.183 (3)
C2—C3	1.403 (3)	C10—H1	0.96 (3)
C3—C4	1.374 (3)		
			
C1—N1—N2	105.49 (15)	C5—C4—H4	120.2
N1—N2—C7	111.24 (15)	C6—C5—C4	124.76 (17)
N1—N2—C8	119.25 (15)	C6—C5—N3	117.59 (15)
C7—N2—C8	129.42 (15)	C4—C5—N3	117.65 (16)
O2—N3—O1	123.55 (17)	C5—C6—C7	114.68 (16)
O2—N3—C5	118.66 (15)	C5—C6—H6	122.7
O1—N3—C5	117.79 (16)	C7—C6—H6	122.7
N1—C1—C2	112.90 (15)	N2—C7—C6	130.79 (16)
N1—C1—Br1	120.03 (14)	N2—C7—C2	106.99 (16)
C2—C1—Br1	127.07 (14)	C6—C7—C2	122.22 (16)
C7—C2—C3	120.67 (17)	N2—C8—C9	112.42 (15)
C7—C2—C1	103.38 (16)	N2—C8—H8A	109.1
C3—C2—C1	135.92 (17)	C9—C8—H8A	109.1
C4—C3—C2	118.00 (17)	N2—C8—H8B	109.1
C4—C3—H3	121.0	C9—C8—H8B	109.1
C2—C3—H3	121.0	H8A—C8—H8B	107.9
C3—C4—C5	119.66 (17)	C10—C9—C8	179.2 (2)
C3—C4—H4	120.2	C9—C10—H1	180 (2)
			
C1—N1—N2—C7	0.35 (19)	O1—N3—C5—C4	−4.7 (2)
C1—N1—N2—C8	177.07 (15)	C4—C5—C6—C7	−0.9 (3)
N2—N1—C1—C2	−0.1 (2)	N3—C5—C6—C7	178.09 (15)
N2—N1—C1—Br1	−179.08 (12)	N1—N2—C7—C6	179.58 (17)
N1—C1—C2—C7	−0.2 (2)	C8—N2—C7—C6	3.3 (3)
Br1—C1—C2—C7	178.71 (13)	N1—N2—C7—C2	−0.49 (19)
N1—C1—C2—C3	−178.39 (19)	C8—N2—C7—C2	−176.79 (17)
Br1—C1—C2—C3	0.5 (3)	C5—C6—C7—N2	−178.79 (17)
C7—C2—C3—C4	0.4 (3)	C5—C6—C7—C2	1.3 (2)
C1—C2—C3—C4	178.4 (2)	C3—C2—C7—N2	178.93 (16)
C2—C3—C4—C5	0.0 (3)	C1—C2—C7—N2	0.41 (18)
C3—C4—C5—C6	0.2 (3)	C3—C2—C7—C6	−1.1 (3)
C3—C4—C5—N3	−178.71 (16)	C1—C2—C7—C6	−179.65 (16)
O2—N3—C5—C6	−4.2 (2)	N1—N2—C8—C9	112.94 (18)
O1—N3—C5—C6	176.26 (16)	C7—N2—C8—C9	−71.0 (2)
O2—N3—C5—C4	174.85 (17)		

### Hydrogen-bond geometry (Å, °)

**Table:**

*D*—H···*A*	*D*—H	H···*A*	*D*···*A*	*D*—H···*A*
C10—H1···O1^i^	0.96 (3)	2.45 (3)	3.399 (3)	167 (3)

Symmetry codes: (i) −*x*+1/2, *y*+3/2, −*z*+1/2.

## References

[bb1] Barbour, L. J. (2001). *J. Supramol. Chem.* **1**, 189–191.

[bb2] Bruker (2009). *APEX2* and *SAINT* Bruker AXS Inc., Madison, Wisconsin, USA.

[bb3] El Brahmi, N., Mohamed, B., Essassi, E. M., Zouihri, H. & Ng, S. W. (2009). *Acta Cryst.* E**65**, o2320. 10.1107/S1600536809034138 PMC2970361 21577791

[bb4] Sheldrick, G. M. (1996). *SADABS* University of Göttingen, Germany.

[bb5] Sheldrick, G. M. (2008). *Acta Cryst.* A**64**, 112–122. 10.1107/S0108767307043930 18156677

[bb6] Westrip, S. P. (2010). *J. Appl. Cryst.* **43**, 920–925.

